# Privacy Leakage in Federated Learning in Radiology Reports: Comparative Evaluation of Tokenizer and Batch-Size Privacy Risks

**DOI:** 10.2196/88390

**Published:** 2026-09-11

**Authors:** Santhosh Parampottupadam, Andrés Martínez Mora, Dimitrios Bounias, Sinem Sav, Klaus Maier-Hein, Ralf Floca

**Affiliations:** 1 Division of Medical Image Computing, German Cancer Research Center, Im Neuenheimer Feld 280, Heidelberg, Baden-Wurttemberg, 69120, Germany, 49 06221 420; 2 Medical Faculty Heidelberg, Heidelberg University, Heidelberg, Germany; 3 Department of Computer Engineering, Bilkent University, Ankara, Türkiye; 4 Pattern Analysis and Learning Group, Department of Radiation Oncology, Heidelberg University Hospital, Heidelberg, Germany

**Keywords:** federated learning, radiology, privacy, gradient inversion, large language models, data security, transformer models, patient confidentiality, Health Insurance Portability and Accountability Act, HIPAA, General Data Protection Regulation, GDPR

## Abstract

**Background:**

Federated learning (FL) enables multi-institutional model training on clinical text without sharing raw data; however, gradient inversion methods can reconstruct sensitive information from shared model updates. The extent of such privacy leakage in FL applied to radiology reports, and the role of tokenizer design, remains unclear.

**Objective:**

This study aimed to quantify gradient-based reconstruction of radiology report text in an FL setting and to compare privacy risk across 3 transformer tokenization strategies in a controlled, tokenizer-aware evaluation.

**Methods:**

Six FL clients trained a GPT-2–style transformer (sequence length 32) on 2 public clinical-text corpora comprising 368,751 diagnostic reports, 98,206 discharge summaries, and 1500 MIMIC-CXR (Medical Information Mart for Intensive Care Chest X-Ray) radiology reports. Models were trained using 3 tokenizers (GPT-2, RadBERT, and LLaMA-2) with batch sizes of 64, 128, and 256. An active malicious-server threat model was assumed, and analytic gradient inversion was applied to recover text. Reconstruction fidelity was measured over 5 runs using exact sentence accuracy, sentence-level bilingual evaluation understudy (S-BLEU), and recall-oriented understudy for gisting evaluation (ROUGE-L).

**Results:**

Exact sentence reconstruction ranged from 27% to 75% across tokenizers, datasets, and batch sizes. At batch size 64 on the discharge dataset, accuracy was 64.7% (GPT-2), 70% (RadBERT), and 67.5% (LLaMA-2), decreasing to 27.3%, 28.5%, and 27.5% at batch size 256. S-BLEU declined with increasing batch size (eg, discharge reports from 0.69 to 0.31). Reconstruction fidelity did not differ significantly across tokenizers (all but one of 27 comparisons nonsignificant; none significant after Holm correction), and approximately 75% of clinical concepts were represented in the reconstructed text (a corpus-level upper bound), regardless of tokenizer. Batch size was the dominant factor governing leakage.

**Conclusions:**

Under a worst-case malicious server that tampers with the shared model and observes unprotected per-client gradients (no secure aggregation or differential privacy), substantial portions of radiology-report text can be reconstructed, with up to approximately 75% of reconstructed 32-token sequences and 75% of clinical concepts (not direct patient identifiers) recovered from FL gradients. In a controlled ablation holding model architecture fixed, tokenizer choice, including domain-specific tokenizers, did not significantly affect leakage under the evaluated conditions, whereas batch size was the primary determinant, and no tokenizer significantly reduced the risk. Tokenizer selection should therefore not be treated as a privacy safeguard in this setting. Safeguards such as secure aggregation and differential privacy should therefore be evaluated as candidate protections for FL deployments that must satisfy Health Insurance Portability and Accountability Act (HIPAA) and General Data Protection Regulation (GDPR) requirements in radiology natural language processing (NLP); legal compliance additionally depends on organizational safeguards, risk assessment, and governance beyond the scope of this study.

## Introduction

### Background

Radiology reports serve as an indispensable tool in medical diagnostics, providing critical insights [1] that complement imaging data. These textual data contain rich clinical information, including patient history, diagnostic impressions, and recommended follow-ups, often bridging gaps left by imaging alone. In recent years, advancements in AI, particularly transformer-based large language models (LLMs) [2], have revolutionized natural language processing (NLP), making it possible to analyze unstructured textual data at scale. LLMs such as the GPT family [3] are now being explored for their potential in radiology to assist in generating summaries [4], extracting relevant clinical insights, and identifying patterns across large datasets [5-7]. A recent systematic review of LLM evaluations in clinical medicine highlights the rapid growth of these models and underscores the need for robust evaluation frameworks to ensure their safety, reliability, and ethical alignment in health care applications [8].

Radiology LLMs can be collaboratively developed across multiple institutions, leveraging diverse clinical reports to enhance model robustness and improve generalizability across unseen health care settings. Nevertheless, health care data, such as radiological data sharing, faces significant challenges due to stringent data protection laws such as the Health Insurance Portability and Accountability Act (HIPAA) [9] in the United States and the General Data Protection Regulation (GDPR) [10] in the European Union, which mandate strict safeguards for the privacy of sensitive patient data. Federated learning (FL) [11], a decentralized machine learning approach where models are trained across multiple institutions without exchanging raw data, has emerged as a promising solution to address these challenges by ensuring that sensitive data remain within each participating institution while still enabling collaborative model development. Multicentric FL clinical collaborations [12-15] are transforming medical research by enabling privacy-preserving data sharing, fostering innovation, and addressing critical concerns around data ownership and security.

Nevertheless, FL’s decentralized nature introduces inherent vulnerabilities that may compromise patient confidentiality [16,17]. While they eliminate direct data sharing, FL systems rely on the exchange of model parameters between institutions and a central server. An active malicious server—one that not only observes gradients but also modifies the shared model architecture before distribution—can exploit these parameters to reconstruct sensitive data, exposing significant vulnerabilities. Existing research has shown that gradient-inversion attacks [18-20] can reconstruct private text from models trained on generic language datasets, but such risks remain largely unexplored in the domain of radiology [21].

Recent advances in NLP have demonstrated that leveraging domain-specific tokenizers [18] and training LLMs on domain-adapted corpora significantly enhances performance in specialized fields such as medicine and genomics. For instance, studies such as Gu et al [22] introduced PubMedBERT, showing that pretraining on biomedical literature yields superior results on downstream clinical tasks compared to generalist models. Similarly, prior studies such as Zhang et al [23] demonstrate that fine-tuning LLMs such as BioBERT and MedAlpaca with domain-specific data and tailored tokenization strategies substantially enhances performance in medical tasks. These adaptations enable the models to capture nuanced, domain-relevant semantics that general-purpose LLMs typically overlook.

To specifically investigate the role of tokenization in privacy leakage within FL, we isolate tokenizer design as the primary variable while keeping the underlying model architecture constant. While most prior work compares entire language models [8], we argue that vocabulary segmentation alone can influence the susceptibility of models to gradient-inversion attacks. Tokenizers trained on domain-specific corpora (eg, RadBERT) are more likely to encode medical terms as single tokens, increasing their semantic coherence; we hypothesized that this would also make such terms easier to reconstruct—a prediction our controlled experiments ultimately did not support (see the “Results” section). In contrast, general-purpose tokenizers tend to fragment clinical phrases into multiple subwords, which may reduce both interpretability and recoverability. This design choice not only impacts downstream clinical utility [22] but might, we hypothesized, also alter the reidentifiability of sensitive entities during model inversion—a prediction our controlled experiments did not support. By decoupling the tokenizer from the model architecture, our approach provides a targeted evaluation of how vocabulary structure relates to the balance between semantic fidelity and patient privacy.

In our controlled evaluation, the radiology-specific RadBERT [24] tokenizer did not yield significantly higher reconstruction fidelity under attack than general-purpose tokenizers such as GPT-2 [25] and LLaMA-2 [26]. This indicates that the advantages of domain-adapted pretraining for capturing clinical semantics do not come at the cost of measurably greater gradient-inversion leakage; the vulnerability is intrinsic to the attack and is governed primarily by batch size—though under this imprint-probe construction the batch-size effect operates proximately through increased bin-collision burden rather than as a general law of federated training. This observation aligns with evidence that tokenizer and model design choices can influence performance in domain-specific applications, raising the question of whether they likewise affect privacy risk under gradient inversion. Building on this insight, we investigate the extent to which radiology report data can be reconstructed from transformer-based models trained in a simulated FL environment. By orchestrating targeted attacks in a multiclient setup using publicly available radiological datasets, we expose critical vulnerabilities in current FL frameworks. Our study deliberately focuses on the attack surface and does not evaluate defense mechanisms or privacy-preserving strategies. Rather, it aims to characterize worst-case risks and motivate future work toward robust, domain-aware privacy protections for safe deployment of clinical foundation models.

### Scope and Contributions

This work establishes a foundational analysis of the privacy vulnerabilities inherent in applying transformer-based FL to radiology reports. We focus on characterizing the attack surface by quantifying the reconstruction risk under a worst-case gradient-inversion scenario. To this end, we provide a systematic comparison of information leakage across major tokenizers, namely, GPT-2, RadBERT, and LLaMA-2. Our central contribution is a controlled demonstration that, with model architecture held fixed, tokenizer choice—including domain-specific tokenizers like RadBERT—does not significantly change the risk of leaking sensitive patient data, whereas batch size does; practitioners therefore should not, in this setting, rely on tokenizer selection as a privacy safeguard. These findings provide a necessary benchmark and guidepost for developing effective privacy-preserving defenses in medical FL systems. Clinical utility is not directly evaluated in this study and is inferred from prior work establishing improved downstream performance with domain-specific clinical tokenizers.

## Methods

### Ethical Considerations

This study is a secondary computational analysis of 2 publicly available, previously deidentified clinical text corpora: the dischargesum dataset [27] and the MIMIC-CXR (Medical Information Mart for Intensive Care Chest X-Ray) free-text radiology reports accessed via PhysioNet [28] under the standard PhysioNet credentialed-access Data Use Agreement. No new data from human participants were collected as part of this work. No identifiable individuals appear in any figure, table, or supplementary material. No participants were recruited, and no compensation was provided. Per institutional policy at the German Cancer Research Center (DKFZ) and consistent with PhysioNet’s terms of use, secondary computational analysis of these deidentified public corpora qualifies for ethics board exemption; the original institutional review board approvals covering the primary collection of MIMIC-CXR (Beth Israel Deaconess Medical Center) and Dischargesum extend to secondary research use without additional consent. All experiments were performed on local DKFZ compute infrastructure and no patient data were transmitted to external services.

### FL and Transformer Models

**Figure 1.**
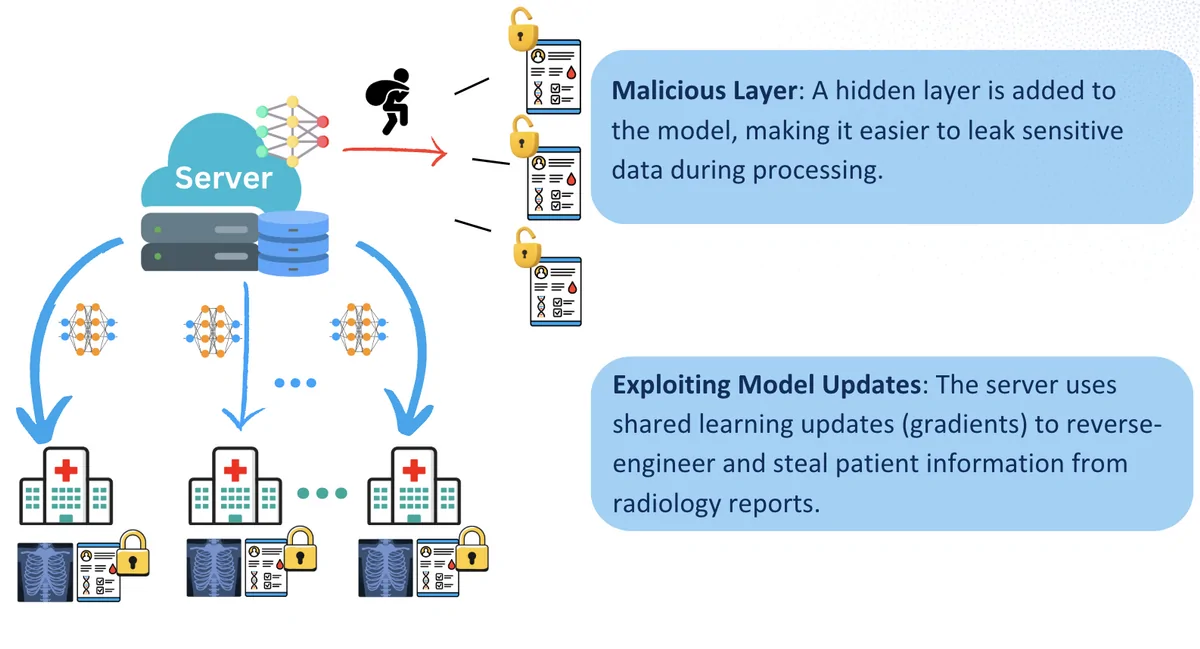
Gradient-inversion attack pathway in federated radiology natural language processing. A malicious central server coordinates model training across multiple hospitals. By inserting a lightweight linear probe at the embedding stage, the server exposes token-level representations and, from the probe’s gradients, reconstructs token embeddings in closed form. Decoding these embeddings via the tokenizer’s embedding table yields patient text, illustrating that federated learning can leak confidential report content even without raw data sharing.

FL [11] is a decentralized machine learning paradigm that allows multiple institutions to collaboratively train a common model without exchanging raw data. Each client trains locally and sends only gradient updates to a central server for aggregation. In our implementation, we simulated this multi-institutional setup using a GPT-2–style transformer (instantiated as a compact 3-layer encoder with token-embedding dimension m=96, 8 attention heads, feed-forward hidden size 1536, and approximately 13.4 M trainable parameters, following the field guide to FL baseline architecture [29]) combined with 3 different tokenizers. The overall attack pathway is illustrated in Figure 1.

- GPT-2 tokenizer: 50,257 tokens
- StanfordAIMI/RadBERT tokenizer: 30,522 tokens
- LLaMA-2-7B tokenizer: 32,000 tokens

All tokenizers operated with a fixed sequence length of 32 tokens, so any observed privacy differences are attributable to vocabulary segmentation rather than to input shape or representational scale. Holding the foundation-model architecture fixed at this small, well-characterized baseline allows the tokenizer to serve as the sole independent variable across our GPT-2, RadBERT, and LLaMA-2 comparisons. The 32-token sequence length follows the field guide to FL baseline configuration [29] and is consistent with prior gradient-inversion experiments on text models [30]; it provides a controlled, computationally tractable regime in which the imprint construction’s bin disentanglement is well-characterized. To emulate real multi-institutional training, we randomly partitioned 2 radiology text datasets across 6 cloud‐based clients: the public Dischargesum corpus (98,206 discharge summaries; 368,751 diagnostic reports) and a locally curated sample of 1500 free-text reports from MIMIC-CXR. Training was repeated with batch sizes of 64, 128, and 256 sequences to measure how aggregation granularity affects privacy leakage in shared gradients. Batch sizes refer throughout to 32-token sequences, not whole reports: each report is tokenized and split into multiple nonoverlapping 32-token windows; a single discharge summary or radiology report typically contributes between 5 and 40 sequences depending on its length, so the per-client local datasets contain ≥5000 sequences and comfortably exceed the largest batch size of 256. Gradient-inversion attacks of the type considered here operate on the gradient produced by a single client during a single training round, which constitutes the standard threat-surface unit in the gradient-leakage literature [30]; privacy leakage was therefore quantified per (client and round) rather than over the aggregated federation-level update.

### Threat Model

#### Overview

We characterize the adversary in this study as an active malicious server, following the worst-case threat-model conventions used in prior FL gradient-inversion analyses [31,32]. Formally, the adversary controls the central FL server and possesses the following capabilities. (C1) Architecture modification (active, white box on the server side): the server may insert auxiliary parameters into the shared model before the first communication round, provided the resulting model still trains end-to-end. We exercise this capability by inserting a single analytic imprint module (see section “Background and Preliminaries”) immediately before the positional embedding. (C2) Per-round per-client gradient observation: the server observes the full set of weight and bias gradients ∇θ ℒ returned by each client at each round. We do not assume access to intermediate activations, hidden states, or any nongradient signal—that is, the protocol is standard FedAvg/FedSGD, not split learning. (C3) Knowledge of the public model and tokenizer: the server knows the model architecture (since it distributed it) and the tokenizer’s embedding table (since it is part of the shared model). The adversary does not observe raw client data, client-side optimization state, or any cryptographic key material. We assume no defenses are enabled at any layer of the FL stack: no differential privacy (DP), no secure aggregation, no client-side architecture verification, no gradient anomaly detection. This intentionally maximal threat model isolates the upper-bound leakage attributable to gradient inversion alone, which is the quantity our experiments seek to characterize.

#### Detectability and Stealthiness

The architecture modification in C1 is in principle detectable by any client that compares the received model’s parameter signature to a published reference. In current production FL frameworks, no such signature verification is mandatory, and small architectural modifications could in principle be disguised by representing the imprint module as a benign feature-extractor or input-normalization layer. However, the imprint module itself adds approximately (1000×96) + (96×1000)+1000≈193 K parameters—roughly 1.4% of the 13.4-million-parameter base model—and would therefore be detectable by any routine client-side parameter-size audit or cryptographic attestation of the published model signature. We therefore treat detectability as a deployment-policy mitigation, not a fundamental defense, and we recommend in section “Practical and Regulatory Implications” that mandatory client-side architecture attestation be considered as part of clinical FL audit protocols.

#### Scope of These Findings

The reported reconstruction rates correspond to a worst-case adversary: a malicious central server that (1) owns and modifies the shared model architecture before distributing it to clients, (2) observes per-round per-client gradients on weight and bias parameters, and (3) operates in a setting with no privacy defenses enabled—no DP, no secure aggregation, no gradient clipping, and no client-side model integrity verification. Under typical real-world cross-silo FL deployments, where institutional clients verify the received model architecture, secure aggregation conceals individual client updates, and lightweight differential-privacy noise is applied, these reconstruction rates would be expected to fall substantially. We therefore interpret our numbers as an upper bound on residual leakage when defenses are absent, not as expected leakage in defended deployments. This framing is consistent with the worst-case methodology of prior gradient-inversion studies [30].

### Background and Preliminaries

Let the token-embedding dimension be m, and the probe output dimension be k. For a single token position, the hidden vector x ∈ ℝ^m^ is produced by the embedding table before positional addition and attention mixing. We insert a small linear probe (an “imprint” module in the sense of [30]) on top of this hidden state:

$y=W_{2}\cdot ReLU\left(W_{0}x+b_{0}\right)$ with W₀ ∈ ℝ^k×m^, b₀ ∈ ℝ^k^, and W₂ ∈ ℝ^m×k^, where k denotes the number of probe bins. The probe is appended once at initialization and trained jointly with the rest of the model. As in standard cross-silo FL (FedAvg [11]), each client transmits only the parameter gradient ∇θ ℒ of its local loss back to the server; no intermediate activations or hidden states are exchanged. This rules out split-learning style leakage and isolates the threat surface to the gradient update itself.

We initialize b₀ to the cumulative quantiles of a Laplace prior over the expected preactivation distribution and W₀ to a random Gaussian matrix; W₂ is initialized so that *y*≈*x* in expectation, leaving downstream training behavior essentially unchanged. Under this construction, the bins partition the input space into cumulative half-spaces of geometrically decreasing measure, so that for sufficiently large k at most one sample in a mini-batch activates each bin after consecutive-bin differencing. In general, batch aggregation destroys per-sample information: the transmitted gradient ∇θ ℒ averages over all B samples in the batch and is not invertible without optimization [30]. The cumulative-bin imprint construction circumvents this barrier by analytic design rather than by optimization—the contribution of each individual token to ∇W₀ and ∇b₀ is structurally separated by bin index i, so the server can isolate per-sample gradients even though it only ever observes the batch sum.

From these 2 captured gradients, the input hidden vector x can be recovered in closed form without optimization: ${\hat{x}}_{j}=\frac{▽W_{0,i}}{▽b_{0,i}}$

where i is the bin uniquely activated by token j after the cumulative-bin differencing step ∇W_₀,i_ ← ∇W_₀,i_ − ∇W_₀,i−1_ (and analogously for ∇b_₀,i_). We apply this per token and per sample in a batch; sentence reconstructions are formed by decoding each $x_{j}$to the nearest token in the active tokenizer’s embedding table E ∈ ℝ^V×m^ under cosine similarity, where V is the tokenizer vocabulary size. We set k=1000 bins and m=96, with $x_{j}$L2-normalized before decoding; when ‖∇b₀,i‖₂ is below a numerical threshold ε, we add a ridge term ε to the denominator for numerical stability.

Ties or near-ties (cosine margin < δ) among candidate tokens are broken by rescoring with the model’s language-model-head logits in a server-side forward pass conditioned on the previously-decoded prefix of the same sample—feasible because the malicious server holds the full shared model and can run forward passes on partial reconstructions. Token j is therefore resolved in left-to-right context rather than in isolation; the very first position falls back to the unconditional logit prior.

### Attack Implementation

#### Overview

We implement the gradient-inversion attack as a deterministic, server-side procedure operating on per-round gradients. The analytic probe and closed-form inversion are defined in the section “Background and Preliminaries”; below we describe the practical instrumentation, data capture, and decoding steps used in our experiments.

#### Step 1: Instrumentation

The server augments the shared transformer with a lightweight linear probe y=Wx+b inserted immediately after the token embedding lookup and before positional additions and attention mixing (see the “Background and Preliminaries” section). This placement exposes token-level representations prior to any token mixing, ensuring reconstructed vectors reflect tokenizer segmentation rather than later architectural effects.

#### Step 2: Gradient Capture

During each client training round, the server records the probe gradients returned in the aggregation step—specifically, the per-round weight gradient ∇W₀ ℒ ∈ ℝ^k×m^ and bias gradient ∇b₀ ℒ ∈ ℝ^k^ of the imprint module’s first linear layer (notation as in “Background and Preliminaries”). The “upstream signal” ∂ℒ/∂y referenced in prior gradient-inversion literature is not transmitted by clients; it is reconstructed server-side from the captured weight and bias gradients under the cumulative-bin construction. These per-round gradients are stored for subsequent inversion and auditing; we retain round identifiers and batch metadata to link reconstructions to training conditions (dataset, tokenizer, and batch size).

#### Step 3: Closed-Form Inversion and Stabilization

For each captured token gradient in a round, we compute the closed-form embedding estimate $\hat{x}$ following the expression in the “Background and Preliminaries” section. After inversion, each $\hat{x}$ is L2-normalized to match embedding norm scaling; when ‖∇b₀,i‖₂ is near zero, we add a small ridge term ε to the denominator for numerical stability (implementation: = ${\hat{x}}_{j}=\frac{▽W_{0,i}}{\left(▽b_{0,i}+\varepsilon \right)},\;\epsilon =10^{-8}$.

#### Step 4: Token Decoding

##### Overview

Each recovered vector $\hat{x}$ is decoded by nearest-neighbor search over the active tokenizer’s embedding table using cosine similarity (top-1). When multiple vocabulary entries are within a small cosine-similarity margin δ of the maximum, the server breaks the tie by rescoring candidates using the model’s language-model-head logit at that position, computed in a server-side forward pass conditioned on the previously-decoded prefix. This is feasible because the malicious server holds the full shared model (it distributed the model in the first place) and can execute forward passes on partial reconstructions; the very first position falls back to the unconditional logit prior. Decoded tokens are concatenated in positional order to yield sentence-level reconstructions.

##### Clarification on Positional Order Recovery

Positional order is determined by an optimal-assignment matching between recovered breached embeddings and the 32 known positional embeddings of the sequence, implemented as scipy.optimize.linear_sum_assignment (the Hungarian algorithm) in the breaching library, following Fowl et al [30]. The language-model-head logit rescoring described above operates within each assigned position to resolve vocabulary ambiguity; it does not itself determine position. The phrase “immediately before the positional embedding” in our previous revision refers to topological placement within the encoder stack (before the attention and token-mixing layers); the recovered breached embeddings nonetheless carry the positional signature used by the matching stage above.

### Step 5: Design for Fair Tokenizer Comparison

#### Overview

To isolate tokenization effects from architecture or training hyperparameters, we hold the model architecture, embedding dimension m, probe output dimension *k*, and probe placement constant across experiments. The same inversion and decoding hyperparameters (ridge ε, L2 normalization, and nearest-neighbor metric) were applied to GPT-2, RadBERT, and LLaMA-2 tokenizer evaluations without per-tokenizer tuning. Because decoding uses each tokenizer’s own embedding table, reconstruction fidelity reflects vocabulary segmentation and tokenizer-induced exposure (single-token entities vs fragmented subwords).

#### Implementation Notes and Novelty

**Figure 2.**
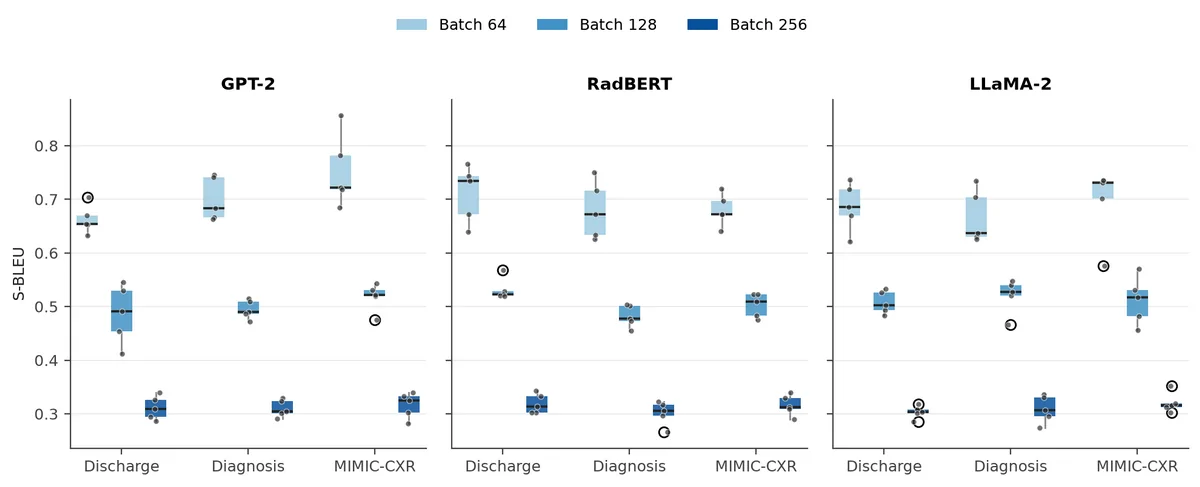
Box plots (with the individual runs overlaid as points) showing the distribution of sentence-level bilingual evaluation understudy scores across 5 runs for each tokenizer (GPT-2, RadBERT, and LLaMA-2) on 3 datasets: discharge, diagnosis, and MIMIC-CXR (Medical Information Mart for Intensive Care Chest X-Ray). Each box represents performance at a given batch size (64, 128, and 256), with color-coded distinctions shown in the legend. The plots illustrate both central tendency and variability, showing that the tokenizer distributions overlap substantially, with no consistent tokenizer advantage in reconstruction fidelity. Distributions compress as batch size increases, reflecting reduced sentence-level recoverability. S-BLEU: sentence-level bilingual evaluation understudy.

All inversion and decoding steps were implemented in Python (NumPy/PyTorch). Nearest-neighbor lookup used a brute-force cosine search for reproducibility; for large vocabularies, the same pipeline is compatible with approximate nearest-neighbor libraries (Facebook AI Similarity Search [FAISS]). Per-round artifacts (timestamps and batch composition) were retained to permit round-level analyses and the box plots reported in Figure 2 and Multimedia Appendices 1 and 2. The analytic probe itself follows prior work [30]; our novelty lies in (1) its embedding-stack placement to isolate tokenizer-specific effects, (2) a tokenizer-controlled, architecture-fixed evaluation showing that tokenizer choice does not significantly change leakage, and (3) clinical entity-level leakage quantification using MedGemma named-entity recognition (NER) [33], which quantifies the recovery of clinical concepts (diagnoses, procedures, anatomy, and medications).

#### Clarification on the Cumulative-Bin Regime

The k=1000 bins are allocated globally over the batch rather than per token position, so the expected per-bin occupancy is (B×T)/k over the full pool of B×T token activations (*t*=32 here). The strict single-occupancy regime (B<k) is therefore not strictly achieved even at our smallest batch: at B=64 the 2048 token activations already exceed the 1000 bins, so a minority of bins are multiply occupied, and multioccupancy grows with batch size until it is pervasive at B=256 (8192 activations). The analytic recovery rule x̂_j_ = ∇W_i_/∇b_i_ is exact only in the single-occupancy regime; under multioccupancy it yields a per-bin weighted average of the active samples’ embeddings, producing a blurred but nonvacuous reconstruction. Because the recovered vector approximates the occupancy-weighted mean of the colliding token embeddings, its nearest neighbor in the embedding table is generally a token near that centroid rather than any single constituent; empirically, this biases decoding toward high-frequency, generic tokens (common subwords and scaffolding words), so specific low-frequency clinical terms are the first to be lost while frequent structural tokens are still recovered—which is why reconstruction degrades gracefully with batch size rather than collapsing to noise. Positional disambiguation in this regime is performed by the optimal-assignment matching stage described in the “Attack Implementation” section (Step 4) operating on the recovered continuous embeddings. The reconstruction-accuracy degradation we report across batch sizes 64 → 128 → 256 is consistent with this graceful degradation rather than a strict pigeonhole failure.

### Dataset and Experimental Setup

#### Overview

We evaluated privacy leakage using 3 clinical-text datasets drawn from 2 public corpora. The Dischargesum [27] dataset contained 98,206 discharge summaries and 368,751 diagnostic reports, which were evenly distributed across 6 simulated institutional clients. To complement these structured reports, we also included a subset of 1500 free-text MIMIC-CXR [28] radiology reports, similarly partitioned across the 6 clients.

#### MIMIC-CXR Sample

From the full MIMIC-CXR free-text corpus (≈227,000 reports), we sampled 1500 reports uniformly at random without replacement, conditional on each report being nonempty after standard whitespace stripping. No filtering was applied by clinical content, report length, institutional source, or temporal range. The sample identifier list is included in our reproducibility package and available from the corresponding author on reasonable request, subject to the PhysioNet Data Use Agreement.

Each client trained a GPT-2–style transformer locally and transmitted only gradient updates to the central server for aggregation, emulating a standard cross-institutional FL workflow. Reports were uniformly sampled at random across the 6 clients, producing an approximately independent and identically distributed (IID) partition for the purposes of this study. Experiments systematically varied the local batch size (64, 128, and 256 sequences) to evaluate its effect on gradient-level privacy leakage. Every configuration was repeated across 5 independent runs to account for stochastic training variability and to ensure reproducibility; 95% CIs across runs are reported in Multimedia Appendix 3 (eg, 64.7%, 95% CI 60.2‐69.2, for GPT-2 on discharge at batch size 64), and a 3-way variance decomposition attributed 94% of the variance in exact-sentence accuracy to batch size versus under 1% to tokenizer; exact paired *t* tests are reported in Multimedia Appendix 4. Training details and reproducibility configuration are reported in Multimedia Appendix 5. At each measurement point, the malicious server captures the gradient transmitted by one client in a given round and applies the analytic reconstruction described in “Background and Preliminaries” to that single update, in line with the standard single-step formulation adopted in the gradient-inversion literature [30]. Each batch-size configuration was evaluated under this per-client, per-round setting across the 5 independent runs.

### Evaluation Metrics

We assessed reconstruction fidelity using 3 complementary metrics chosen to capture both exact and partial text recovery, following established evaluation practice for reconstructed clinical text [14-17,24]. These metrics together quantify the extent to which patient-identifiable information can be recreated from shared model updates.

- Exact sentence accuracy: The percentage of reconstructed 32-token sequence windows—the fixed-length units into which reports are tokenized (see the “Threat Model” section), for which “sentence” is used as shorthand—that match the ground-truth window exactly, reflecting the proportion of complete exposures of patient text. A reconstructed sentence is counted as an exact match if, after the following normalization steps, every token at every position equals the corresponding token in the original sentence: (1) unicode NFKC normalization, (2) lower-casing, (3) collapsing all runs of internal whitespace to a single space, and (4) preserving punctuation as part of the token sequence rather than stripping it. Sentences are extracted from the concatenated stream of decoded 32-token windows by segmenting at terminal punctuation; the metric is therefore evaluated on these reconstructed sentence units rather than directly on the raw 32-token chunks. We report a stricter case-sensitive variant on the same 5-run experiment for completeness; the qualitative ordering of tokenizers is unchanged across both variants. Exact-sentence accuracy is reported as the conservative upper bound on full-sentence exposure: token-level partial recovery (a single recovered diagnostic noun phrase, for instance) can constitute clinically meaningful leakage even when the full sentence is not reconstructed exactly; this regime is captured by the n-gram and longest-common-subsequence metrics described below. The two metric families together bracket the true leakage rate.
- Sentence-level bilingual evaluation understudy (S-BLEU) [34,35]: Calculates n-gram precision, where an n-gram is a contiguous sequence of n words (eg, unigrams, bigrams, trigrams, and 4-grams). Bilingual evaluation understudy (BLEU) compares 1‐4 word sequences in the reconstructed sentence against the reference, with a brevity penalty to discourage overly short outputs. Higher scores indicate stronger local overlap and preservation of clinical phrasing.
- Recall-oriented understudy for gisting evaluation (ROUGE-L) [36]: Measures recall based on the longest common subsequence (LCS) between reconstruction and reference, capturing the extent to which key clinical terms and their order are retained in longer spans.

By combining these 3 metrics, we quantify both the frequency of perfect reconstructions and the degree of partial text recovery, offering a comprehensive assessment of patient data exposure under our FL attack.

### Choice of Metrics

BLEU and ROUGE-L were originally proposed for translation and summarization quality, where their numerical values are bounded by human-level n-gram overlap. In the gradient-inversion context, we use them as information-recovery metrics rather than as quality metrics: a higher BLEU or ROUGE-L between a reconstructed sentence and the original indicates more shared n-gram content—that is, a greater amount of original wording recovered. Exact-sentence accuracy provides the strict-recovery upper bound; S-BLEU and ROUGE-L provide the partial-recovery signal that captures cases where most of a sentence is recovered but a small number of tokens are misdecoded. This complementary use of strict and partial metrics is consistent with prior gradient-inversion text studies [37]. We deliberately do not adopt semantic-similarity metrics such as BERTScore in the main results because they can credit paraphrastic matches that do not constitute literal information leakage, which is the privacy question of interest here.

### Named-Entity Reference-Vocabulary Overlap Evaluation

#### Overview

We quantified recovery of clinically meaningful content using the Google MedGemma medical NER model [33]. MedGemma was run on the original reports in each dataset to build a per-dataset reference vocabulary of unique clinical entity surface forms (diagnoses, procedures, anatomical structures, and medications). The same model was applied to the reconstructed text for each tokenizer configuration and dataset.

#### Normalization and Matching

Entity strings were lowercased and whitespace-trimmed; internal punctuation was preserved. An entity was counted as recovered if its surface form exactly matched any term in that dataset’s reference vocabulary (case-insensitive). Because this is a corpus-level criterion, it measures reference-vocabulary overlap and represents an upper bound on identifiable leakage: it confirms that a clinical term was reconstructed within the batch but does not by itself establish alignment to a specific source report or patient. No fuzzy matching, synonym expansion, or manual adjudication was used.

#### Metric

For each tokenizer and dataset, we report reference-vocabulary overlap (%), defined as the percentage of the dataset’s MedGemma-extracted reference clinical-concept vocabulary that appears in the reconstructed text, averaged over 5 random seeds (mean and SD); this is a corpus-level recall measure and is not aligned to the specific source report. We report per-dataset and pooled overlap across datasets.

## Results

### Quantitative Reconstruction Success

On the discharge dataset, exact sentence reconstruction accuracy across tokenizers and batch sizes was as follows:

- GPT-2 tokenizer: 64.7%/46.4%/27.3% at batch sizes 64/128/256
- RadBERT tokenizer: 70%/50.8%/28.5%
- LLaMA-2 tokenizer: 67.5%/48.4%/ 27.5%

**Figure 3.**
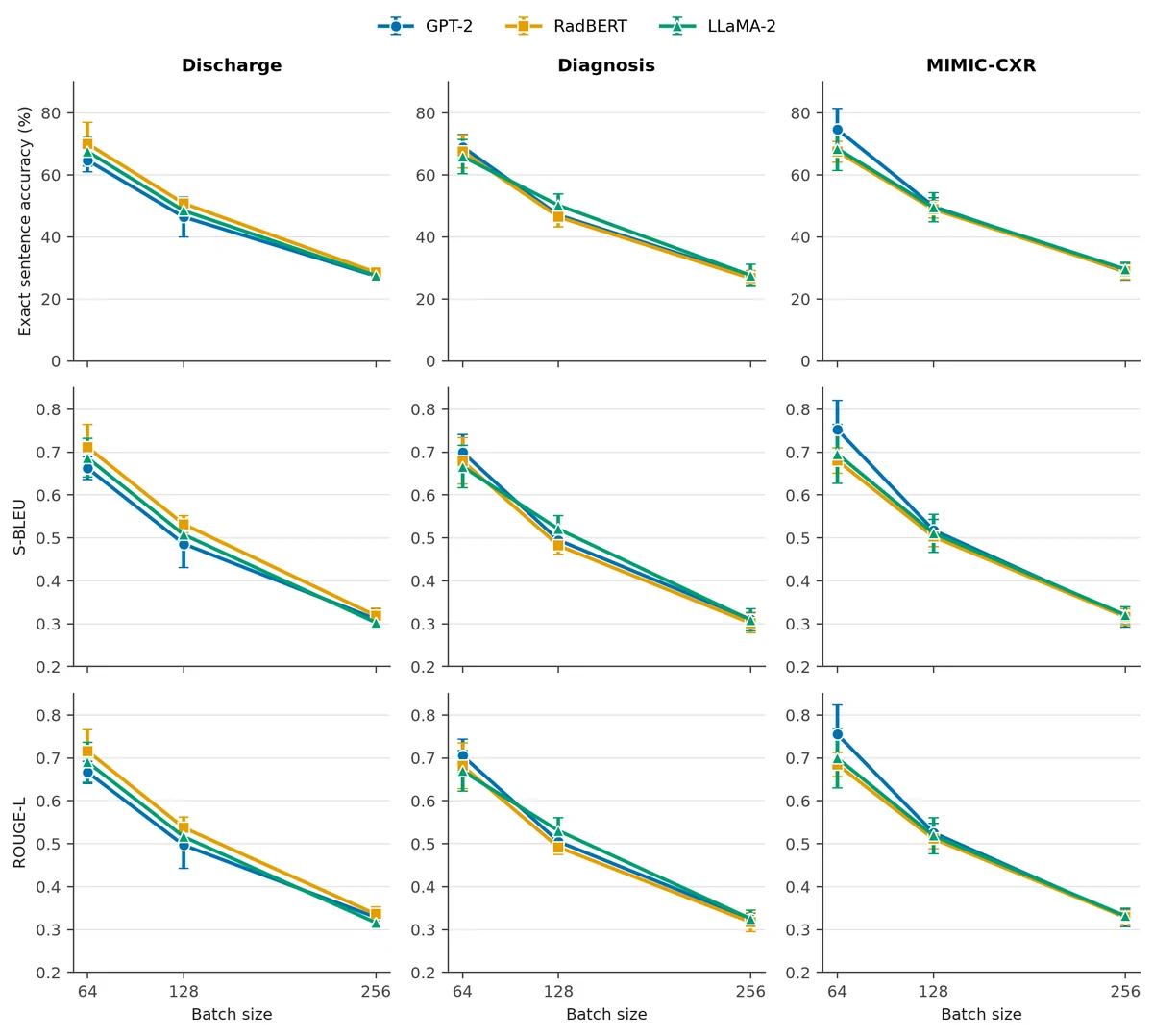
Reconstruction fidelity across datasets and tokenizers at varying batch sizes. Top row: exact sentence reconstruction accuracy; middle row: sentence-level bilingual evaluation understudy scores; bottom row: ROUGE-L scores. Each column corresponds to a dataset (discharge, diagnosis, and MIMIC-CXR [Medical Information Mart for Intensive Care Chest X-Ray]), and colors indicate tokenizers (GPT-2, RadBERT, and LLaMA-2). Across metrics and datasets, reconstruction fidelity is comparable among the 3 tokenizers, with differences within seed-to-seed variability. Increasing the batch size from 64 to 256 reduces reconstruction quality across all models, illustrating a trade-off between training granularity and patient data privacy. S-BLEU: sentence-level bilingual evaluation understudy.

On MIMIC-CXR, reconstruction ranged from 28.7% to 74.7% of sentences (Figure 3), showing that neither tokenizer choice nor larger batch sizes fully mitigates data leakage. Detailed accuracy results are provided in Multimedia Appendix 6. All reported reconstruction metrics represent the mean of 5 independent runs; 95% CIs across runs are reported in Multimedia Appendix 3; exact paired *t* tests are reported in Multimedia Appendix 4. Across configurations, per-cell SDs ranged from approximately 1.5 to 7 percentage points.

### Impact of Batch Size on S-BLEU

Increasing the batch size led to a clear reduction in average reconstruction fidelity across all metrics (Multimedia Appendix 7; Figure 2). For discharge reports, mean S-BLEU decreased from 0.69 at a batch size of 64 to 0.31 at 256; diagnosis reports fell from 0.68 to 0.31; and MIMIC-CXR from 0.71 to 0.32. Parallel trends were observed for ROUGE-L, confirming that coarser gradient aggregation generally mitigates privacy leakage. Notably, across datasets and batch sizes, the 3 tokenizers achieved statistically indistinguishable reconstruction scores. This indicates that domain-specific tokenization does not measurably facilitate more accurate recovery of clinical language, and that tokenizer choice is not a privacy-relevant design lever in this setting. Corresponding mean S-BLEU and ROUGE-L scores are summarized in Multimedia Appendix 7.

### Variability in Gradient-Inversion Severity Across Training Rounds

While average reconstruction fidelity declined with larger batch sizes across all metrics, individual training rounds revealed substantial fluctuations in leakage severity. We illustrate this variability using S-BLEU in Figure 2, as it provides a balanced measure of local n-gram precision and sentence-level structure, making it particularly sensitive to round-to-round differences. However, similar variability patterns were also observed for exact sentence reconstruction accuracy and ROUGE-L, which are summarized in Multimedia Appendices 1 and 2. Even at the highest batch size (256), certain rounds produced high-fidelity reconstructions comparable to those at batch size 64, underscoring a persistent privacy risk. Coarser gradient aggregation therefore reduces but does not eliminate patient information exposure in FL.

In real-world medical AI deployments, even a single outlier round with high leakage could compromise patient confidentiality. Therefore, it is essential to characterize not just average-case leakage but also variability across rounds, to ensure that system-level guarantees account for worst-case scenarios.

### Clinical-Concept Reference-Vocabulary Overlap in Reconstructed Text

As presented in Table 1, reference-vocabulary overlap was high and statistically indistinguishable across tokenizers: MedGemma-extracted clinical-concept overlap averaged 75.1% (GPT-2), 74.1% (RadBERT), and 72.9% (LLaMA-2) across datasets and seeds, with no pairwise difference reaching significance.

These findings demonstrate that gradient-inversion attacks can reveal not only structural sentence fragments but also clinically meaningful entities, posing a tangible privacy risk. That overlap remains high (approximately 75%) regardless of the tokenizer indicates that this leakage is intrinsic to the attack rather than a property of any particular tokenizer in federated training settings.

**Table 1.** Recovery of clinically meaningful terms from gradient-inverted reconstructions using GPT-2, LLaMA-2, and RadBERT tokenizers^a^.

Tokenizer	Discharge, mean (SD)	Diagnosis, mean (SD)	MIMIC-CXR^b^, mean (SD)	Pooled, mean (SD)
GPT-2	71.0 (5.7)	73.0 (3.5)	81.3 (7.1)	75.1 (7.0)
RadBERT	73.7 (5.8)	72.4 (7.9)	76.3 (7.6)	74.1 (6.8)
LLaMA-2	71.8 (7.6)	72.6 (3.0)	74.2 (10.1)	72.9 (7.0)

a Clinical named entities were identified via the Google MedGemma model across all 3 datasets and 5 random seeds. Values are clinical-concept reference-vocabulary overlap (%)—the percentage of each dataset’s reference clinical-concept vocabulary appearing in reconstructions, a corpus-level recall measure—mean (SD); differences across tokenizers are not statistically significant. Pooled pairwise comparisons (n=15): RadBERT vs GPT-2 Δ=−1.0 pp; RadBERT vs LLaMA-2 *Δ*=+1.2 pp; GPT-2 vs LLaMA-2 *Δ*=+2.2 pp—none statistically significant.

b MIMIC-CXR: Medical Information Mart for Intensive Care Chest X-Ray.

### Qualitative Reconstruction

Tables 2–4 showcase original and reconstructed outputs from the dischargesum, radiology, and MIMIC-CXR datasets, illustrating the clinical content recoverable across all 3 tokenizers (aggregate metrics show no significant tokenizer difference). These examples use oracle alignment for readability only: the positional ordering shown is not available to the attacker, who observes an unordered or partially ordered set of recovered tokens, so the aligned display illustrates recoverable content rather than an attacker’s verbatim output. Across the reconstructions, all 3 tokenizers recovered substantial clinical content while also dropping or fragmenting individual terms; in the specific example shown, recovered entities included terms such as “catheter” and “nodularity.” Which terms were retained or dropped varied from example to example, and—consistent with the aggregate analysis, which found no significant tokenizer difference—these single-example observations should not be read as a systematic tokenizer ordering. These qualitative results reinforce the quantitative findings, demonstrating that even domain-adapted transformer models remain susceptible to gradient-based inversion attacks, recovering not only template phrases but also clinically meaningful patient information.

**Table 2.** Discharge report: reconstruction across tokenizers (GPT-2, LLaMA-2, and StanfordAIMI/RadBERT; Oracle-aligned visualization for readability; not attacker-observable output)^a^.

Discharge report: reconstruction across tokenizers	Sample Report Text
Original report	Dear Mr. Alex, It Was A Pleasure Taking Care Of You Here At ABC clinic. You Were Admitted To Our Hospital After Undergoing Repair Of Your Ventral Hernia. You Have Recovered From Surgery And Are Now Ready To Be Discharged To Home With Services. Please Follow The Recommendations Below To Ensure A Speedy And Uneventful Recovery. ACTIVITY: - Do not drive until you have stopped taking pain medicine and feel you could respond in an emergency. - You may climb stairs. - You may go outside, but avoid traveling long distances until you see your surgeon at your next visit.
GPT-2	Dear Mr. Alex, It Was A [DROP] Taking Care Of You [DROP] At ABC clinic. You Were Admitted To Our Hospital After Undergoing Repair Of Your Ventral Hernia. You Have Recovered From Surgery And Are Now Ready To Be Discharged To Home With Services. Please Follow The Recommendations [DROP] To Ensure A And [DROP] Recovery. ACTIVITY: - Do [DROP] drive until you have [DROP] taking pain medicine and feel you could respond in an emergency. - You may climb stairs. - You may go outside, but avoid traveling [DROP] distances until you [DROP] your surgeon at your next visit.
LLaMA-2	[DROP] Mr. Alex, It [DROP] A Pleasure Taking Care Of You Here At ABC clinic. You Were Admitted To Our Hospital After Undergoing Repair Of Your Ventral [DROP]. You Have [DROP] From [DROP] And [DROP] [DROP] [DROP] To Be Discharged To Home With Services. Please Follow The [DROP] Below To Ensure A Speedy And Uneventful Recovery. ACTIVITY: - Do not drive until you have stopped taking pain [DROP] and feel you [DROP] [DROP] in an emergency. - [DROP] [DROP] climb stairs. - You may go outside, but avoid traveling long distances until you see your surgeon at your next visit.
StanfordAIMI/RadBERT	Dear Mr. Alex, It Was A [DROP] Taking Care Of You Here At ABC clinic. You Were Admitted To Our Hospital After Undergoing Repair Of Your Ventral Hernia. You Have Recovered From [DROP] And Are Now Ready To Be Discharged To Home With Services. Please Follow The Recommendations Below To Ensure A Speedy And Uneventful Recovery. ACTIVITY: - Do not drive until you have stopped taking pain medicine and feel you could [DROP] in an [DROP]. - You may climb stairs. - You may go outside, but avoid traveling [DROP] [DROP] until you see your [DROP] at your next visit.

a The original report and reconstructions are aligned by model. Recovered content varies across models in this single example; aggregate reconstruction metrics do not differ significantly across tokenizers (Figure 3; Table 1). The [DROP] marker indicates unreconstructed spans (native source redactions in Table 3 are preserved as asterisks). Note on alignment. The [DROP] marker indicates token positions for which the gradient-inverted reconstruction did not produce a valid token (a bin collision or numerical-instability bin). For visual clarity, these positions are aligned against the original text using oracle alignment, which the attacker does not have access to in a real attack setting; the attacker would observe an unordered or partially ordered set of recovered tokens without knowledge of where reconstruction failed. Oracle alignment is used here purely for reader interpretability and does not reflect adversary capability.

**Table 3.** Radiology report: comparison of reconstructed output across tokenizers; Oracle-aligned visualization for readability; not attacker-observable output)^a^.

Radiology report: reconstruction across tokenizers	Sample Report Text
Original report	EXAMINATION: LIVER OR GALLBLADDER US (SINGLE ORGAN) INDICATION: History: with cirrhosis, increased abdominal pain TECHNIQUE: Gray scale and color Doppler ultrasound images of the right upper quadrant were obtained. COMPARISON: Abdominal ultrasound from **** FINDINGS: The liver is extremely coarse and nodular in echotexture similar to the prior examination consistent with a history of cirrhosis. Parenchymal heterogeneity limits detection of focal lesions.
GPT-2	[DROP]: LIVER OR GALLBLADDER US (SINGLE ORGAN) INDICATION: History: with [DROP], increased abdominal pain TECHNIQUE: Gray scale and color [DROP] ultrasound images of the right upper quadrant were obtained. COMPARISON: Abdominal ultrasound from **** FINDINGS: The liver is [DROP] coarse and [DROP] in echotexture similar to the prior examination consistent with a history of [DROP]. Parenchymal heterogeneity limits detection of focal [DROP].
LLaMA-2	EXAMINATION: [DROP] OR GALLBLADDER US ([DROP] ORGAN) [DROP]: History: with cirrhosis, [DROP] abdominal pain TECHNIQUE: Gray scale and color [DROP] ultrasound images of the right upper quadrant were obtained. COMPARISON: Abdominal ultrasound from **** FINDINGS: The liver is extremely [DROP] and nodular in [DROP] similar to [DROP] prior examination consistent with a history of cirrhosis. Parenchymal [DROP] limits detection of focal lesions.
StanfordAIMI/RadBERT	EXAMINATION: LIVER OR GALLBLADDER US (SINGLE ORGAN) INDICATION: History: with cirrhosis, increased abdominal pain TECHNIQUE: Gray scale and color [DROP] ultrasound images of the right upper quadrant were obtained. [DROP]: Abdominal ultrasound from **** FINDINGS: The liver is extremely [DROP] and nodular in echotexture similar to the prior examination consistent with a history of cirrhosis. [DROP] heterogeneity limits detection of focal lesions.

a In this single example, RadBERT and GPT-2 preserved more contextual phrasing than LLaMA-2; aggregate metrics show no significant tokenizer difference (Figure 3; Table 1). Sensitive findings such as ”cirrhosis” and ”nodularity” were partially reconstructed in all models. Note on alignment. The [DROP] marker indicates token positions for which the gradient-inverted reconstruction did not produce a valid token (a bin collision or numerical-instability bin); 4-asterisk sequences (****) in the Original report row are preserved as native source deidentification redactions from the discharge or radiology reports. For visual clarity, these positions are aligned against the original text using oracle alignment, which the attacker does not have access to in a real attack setting; the attacker would observe an unordered or partially ordered set of recovered tokens without knowledge of where reconstruction failed. Oracle alignment is used here purely for reader interpretability and does not reflect adversary capability.

**Table 4.** MIMIC-CXR^a^ report: reconstruction outputs across tokenizers; Oracle-aligned visualization for readability; not attacker-observable output)^b^.

MIMIC-CXR report: reconstruction across tokenizers	Sample Report Text
Original report	10439781,55811525, “Frontal and lateral views of the chest were obtained. Left-sided Port-A-Catheter is similar in position, terminating at the cavoatrial/right atrial junction. Patient has diffuse increase in interstitial markings bilaterally consistent with patient’s underlying history of chronic interstitial lung disease with likely overlying pulmonary edema improved since ___, but similar in appearance as compared to ___. No definite focal consolidation or pleural effusion. Multilevel vertebroplasties are seen along the thoracic spine, similar to prior.,”Pulmonary edema superimposed on known lung fibrosis.
GPT-2	10439781,55811525, “Frontal and [DROP] views of the chest were obtained. Left-sided Port-A-[DROP] is similar in position, terminating at the cavoatrial/right atrial junction. Patient has diffuse increase in interstitial markings bilaterally consistent with patient’s underlying history of chronic [DROP] lung disease [DROP] likely overlying pulmonary edema improved since ___, but similar in [DROP] as compared to ___. No definite focal consolidation or [DROP] effusion. Multilevel vertebroplasties are seen along the thoracic spine, [DROP] to prior.,”Pulmonary edema superimposed on known lung fibrosis.
LLaMA-2	10439781,[DROP], “Frontal and lateral [DROP] of the chest were [DROP]. Left-sided Port-A-Catheter is similar in position, terminating at the cavoatrial/right atrial junction. Patient has diffuse increase in interstitial markings [DROP] consistent with patient’s underlying history of chronic interstitial lung [DROP] with likely [DROP] pulmonary edema improved since ___, but similar in [DROP] as compared to ___. No [DROP] focal consolidation or pleural [DROP]. Multilevel vertebroplasties are seen along the thoracic spine, similar to prior.,”Pulmonary edema superimposed on known lung [DROP].
StanfordAIMI/RadBERT	10439781,55811525, “Frontal and [DROP] views of the chest were obtained. Left-sided Port-A-Catheter is similar in position, terminating at the cavoatrial/right atrial junction. Patient has diffuse increase in interstitial markings bilaterally consistent with patient’s underlying history of chronic interstitial lung disease with likely overlying pulmonary edema improved since ___, but similar in appearance as compared to ___. No [DROP] focal consolidation or pleural effusion. [DROP] vertebroplasties are seen along the thoracic spine, similar to prior.,”Pulmonary edema [DROP] on known lung fibrosis.

a MIMIC-CXR: Medical Information Mart for Intensive Care Chest X-Ray.

b GPT-2 and RadBERT captured anatomical and pathological keywords such as “fibrosis” and “catheter” more faithfully than LLaMA-2. In this single example, structural fidelity was higher in the RadBERT output; aggregate metrics show no significant tokenizer difference (Figure 3; Table 1). Note on alignment. The [DROP] marker indicates token positions for which the gradient-inverted reconstruction did not produce a valid token (a bin collision or numerical-instability bin). For visual clarity, these positions are aligned against the original text using oracle alignment, which the attacker does not have access to in a real attack setting; the attacker would observe an unordered or partially ordered set of recovered tokens without knowledge of where reconstruction failed. Oracle alignment is used here purely for reader interpretability and does not reflect adversary capability.

## Discussion

### Principal Results

This study demonstrates that even with domain-specific tokenization and larger batch sizes, transformer models in an FL setup leak substantial patient information. The active server reconstructed up to approximately 75% of radiology-report sentences. On the Discharge dataset, exact-sentence accuracy was 64.7%/46.4%/27.3% for GPT-2, 70%/50.8%/28.5% for RadBERT, and 67.5%/48.4%/27.5% for LLaMA-2 at batch sizes 64/128/256. S-BLEU similarly declined with batch size (eg, GPT-2 on discharge reports from 0.66 to 0.31), yet even at the largest batch sizes roughly a quarter of sentences were fully recovered. Across batch sizes and datasets, reconstruction fidelity did not differ significantly among the 3 tokenizers (mean exact-sentence accuracy 48.3% for each; no pairwise comparison significant). After Holm correction for the 27 pairwise comparisons, no tokenizer difference remained significant (all adjusted *P*=1.00), and a 3-way variance decomposition attributed 94% of the variance in exact-sentence accuracy to batch size versus less than 1% to tokenizer, corroborating batch size—not tokenizer choice—as the dominant factor; the pairwise tokenizer effect sizes were negligible (mean differences ≤0.05 percentage points; Cohen *d*≤0.03). All 3 tokenizers, including the domain-specific RadBERT, leaked substantial clinical content, and none eliminated leakage. These findings confirm that neither increasing batch sizes nor specialized tokenizers alone can ensure patient privacy in FL‐trained LLMs.

To further disambiguate templated language from truly clinical leakage, we applied MedGemma NER to the reconstructed text across all 3 datasets. Across models, approximately 75% of reference clinical entities were recovered (Table 1). These findings confirm that privacy risks extend beyond boilerplate phrasing to include clinical concepts relevant to diagnosis and care.

Our findings underscore that tokenizer design is not a decisive factor in privacy leakage under gradient inversion attacks. In a controlled named-entity analysis, clinical-concept reference-vocabulary overlap was statistically indistinguishable across tokenizers, with approximately 75% of each dataset’s reference vocabulary appearing under each (GPT-2 75.1%, RadBERT 74.1%, LLaMA-2 72.9%; no pairwise difference significant). This indicates that domain-specific vocabulary segmentation does not, by itself, make reidentification-relevant clinical content easier to reconstruct. Within the evaluated architecture, attack, datasets, and metrics, tokenizer choice should therefore not be treated as a privacy safeguard; batch size and explicit defenses are the privacy-relevant levers. Future privacy audits and risk assessments of federated clinical models should focus on batch size and explicit defenses rather than tokenizer choice, especially as domain-specific LLMs become more prevalent in medical AI.

### Hypothesized Mechanism and Why It Is Not Borne Out

We initially reasoned that domain-specific tokenization should heighten reconstruction. The reconstruction step decodes each recovered token-embedding vector back to the nearest token in the active tokenizer’s embedding table. A clinical concept such as “pneumothorax” is encoded as a single token in RadBERT’s domain-specific vocabulary, so a successful nearest-neighbor decode would yield the entire concept verbatim, whereas the same concept is segmented into multiple subwords (eg, “p/neu/moth/orax”) in GPT-2’s byte-pair encoding, where recovering it requires every subword embedding to be reconstructed correctly and placed in the correct positional order. Under this reasoning, single-token clinical terms should present fewer points of failure. We tested this prediction directly—including a prespecified analysis restricted to clinical terms that GPT-2 fragments into multiple subwords, where any single-token advantage should be largest—and found no significant tokenizer difference in exact-sentence accuracy, clinical-concept overlap, or the fragmented-term subset. The hypothesized single-token advantage is therefore not borne out empirically: across the datasets and batch sizes studied, the closed-form attack recovers clinical content at comparable rates regardless of tokenizer, and batch size is the dominant factor. This controlled negative result indicates that, in this setting, tokenizer selection should not be treated as a privacy safeguard.

### Clinical Concepts Versus Directly Identifiable Protected Health Information

The MedGemma NER model used in this study captures clinical concepts (diagnoses, procedures, anatomical structures, and medications) rather than direct identifiers under the HIPAA Safe Harbor enumeration (names, medical record numbers, dates more granular than year, full-face photographic images, and so on). Direct identifiers are largely already redacted in the public Dischargesum and MIMIC-CXR corpora before release. The recovered terms therefore reflect reidentification potential through clinical context—for example, an unusually rare diagnosis, a unique procedure pattern, or a specific medication regimen that, in combination with auxiliary recovered context, could plausibly contribute to reidentifying a patient—rather than direct exposure of an enumerated identifier. We make this distinction explicit because the regulatory framing of “PHI” is broader than “direct identifier”; HIPAA’s expert-determination standard (45 CFR §164.514(b)(1)) treats clinical context recoverable from a record as part of identifiability risk, and the same logic is reflected in the GDPR’s notion of indirect identifiability via singling out.

### Why Batch Size Reduces Leakage in This Attack

The imprint module’s analytic recovery rule x̂_j_ =∇W_₀,i_/∇b_₀,i_ relies on the assumption that bin i is activated by at most one sample in the batch. With k=1000 bins and batch size B, the expected number of samples per bin is B/k, and the probability that any given bin is hit by ≥2 samples grows approximately quadratically in B (a birthday-paradox argument). When 2 or more samples share a bin, the recovered ratio ∇W_₀,i_/∇b_₀,i_ becomes a gradient-weighted average of distinct embeddings rather than a clean single-sample recovery, and the corresponding nearest-neighbor decode is corrupted. This explains why exact-sentence accuracy decays with batch size while never reaching zero: as long as some bins remain singly occupied, the corresponding samples are recovered cleanly. The same argument predicts that increasing k relative to B would partially compensate for batch growth—a direction we leave to future work.

### Key Contribution

We provide the first systematic evaluation of how different transformer tokenizers impact gradient‐inversion vulnerability on radiology text, showing that domain‐specific tokenization (RadBERT) does not significantly change leakage risk relative to general-purpose tokenizers, even as it better preserves clinical terminology, whereas batch size does.

### Comparison With Prior Work

#### Overview

Our results extend prior demonstrations of gradient-inversion attacks in generic NLP models [32,38] and imaging domains [20] to the structured and template-rich text of radiology reports. Earlier works such as DLG (deep leakage from gradients) [38] and iDLG (improved deep leakage from gradients) [39] reconstructed generic sentences or pixel-level images from shared gradients but did not examine how tokenizer design mediates privacy leakage. Subsequent privacy-preserving FL frameworks in medical imaging [18-20] primarily focused on architectural defenses rather than language-specific vulnerabilities. Unlike these approaches, our study isolates the tokenizer as a controllable privacy variable by holding the transformer architecture and training hyperparameters constant across GPT-2, RadBERT, and LLaMA-2. This tokenizer-controlled setup reveals that vocabulary segmentation alone does not significantly influence gradient-based reconstruction fidelity. This comparison against prior gradient-inversion literature is summarized in Table 5. While Akinci et al [21] first discussed text leakage risks in clinical LLMs, no prior work systematically compared multiple tokenization schemes or evaluated reconstruction across both discharge and imaging-report corpora. Our findings therefore provide the first controlled evidence that domain-adapted tokenizers, though beneficial for clinical utility, do not measurably reduce or amplify privacy leakage in federated radiology LLMs; batch size, not tokenizer choice, governs reconstruction risk.

**Table 5.** Comparison with prior gradient-inversion studies. To our knowledge, this is the first study to explicitly hold the foundation-model architecture constant and vary only the tokenizer, in the clinical radiology NLP^a^ domain.

Study	Model	Tokenizer controlled?	Domain	Datasets	Defense evaluation?
DLG^b^	LSTM^c^, ResNet^d^	No (single tokenizer per task)	Generic vision + NLP	CIFAR^e^, MNIST^f^	None
iDLG^g^	LeNet	No	Generic vision	CIFAR	None
TAG	BERT^h^	Single tokenizer	Generic NLP	CoLA^i^, SST-2^j^	None
Robbing the Fed	Transformer/ViT^k^	No	Vision + WikiText	WikiText, ImageNet	DP^l^ discussed
Hatamizadeh et al [20]	UNet, ViT	N/A^m^ (vision)	Medical imaging	BraTS^n^, LIDC^o^	Discussed
Akinci et al [21]	Generic LLMs^p^	Discussed, not measured	Clinical NLP	N/A (review article)	—^q^
This work	3-layer Transformer (held constant)	Yes — three tokenizers compared	Clinical radiology NLP	Dischargesum, MIMIC-CXR^r^	Discussed; full evaluation noted as future work

a NLP: natural language processing.

b DLG: deep leakage from gradients.

c LSTM: long short-term memory.

d ResNet: residual network.

e CIFAR: Canadian Institute for Advanced Research.

f MNIST: Modified National Institute of Standards and Technology database.

g iDLG: improved deep leakage from gradients.

h BERT: bidirectional encoder representations from transformers.

i CoLA: Corpus of Linguistic Acceptability.

j SST-2: Stanford Sentiment Treebank (binary).

k ViT: vision transformer.

l DP: differential privacy.

m N/A: not applicable.

n BraTS: Brain Tumor Segmentation (challenge).

o LIDC: Lung Image Database Consortium.

p LLM: large language model.

q Not specified.

r MIMIC-CXR: Medical Information Mart for Intensive Care – Chest X-Ray.

#### Practical Significance

Earlier closed-form gradient inversion attacks on text models report exact-sentence recovery rates of approximately 30%‐50% at small batch sizes on generic English corpora [30,37]; our radiology-domain experiments reach comparable and, at small batch sizes, higher rates (27%‐75%), confirming that the threat extends from generic to clinical NLP and is, if anything, more severe at the strict-recovery level. The practical significance is, however, qualitatively different in the clinical setting: radiology corpora encode clinical context (diagnoses, procedure types, and anatomical findings) at higher density per token than newswire or web text, so equivalent reconstruction rates translate to higher clinical informativeness per recovered sentence. A single recovered sentence in a clinical context may contain a rare diagnosis or unusual procedure combination that contributes meaningfully to reidentification risk, whereas a single recovered sentence from generic web text typically carries no analogous reidentifying signal.

#### Scope of the Tokenizer Comparison

Because we hold the foundation-model architecture fixed at a small, well-characterized baseline, our findings reflect the effect of vocabulary segmentation on gradient-inversion leakage, not the privacy properties of the GPT-2, RadBERT, or LLaMA-2 foundation models themselves. Larger frontier-scale foundation models exhibit different gradient sparsity and noise dynamics that can change inversion vulnerability in either direction; we do not claim that our results extend to those regimes, and explicit characterization at frontier scale is an important target for future work.

### Practical and Regulatory Implications

#### Focus on Attack Characterization

This study deliberately isolates the gradient‐inversion attack vector and does not evaluate or compare privacy defenses. Instead, we quantify the worst-case leakage risk under an unconstrained adversary. Subsequent work should build on these findings to empirically evaluate and optimize defense mechanisms in realistic radiology FL pipelines. Our results demonstrate that generic FL defenses such as DP [40], secure aggregation [41], and gradient‐anomaly detection have yet to be empirically validated for the structured, high‐risk text found in radiology reports. Injecting noise via DP may reduce reconstruction fidelity but requires careful calibration to avoid degrading critical diagnostic language. Similarly, cryptographic secure aggregation can obscure individual updates but may introduce prohibitive latency in hospital networks. Real‐time gradient‐anomaly detection promises early warning of inversion attacks, yet its thresholds must be tuned to the unique update patterns of clinical text to prevent both false alarms and missed breaches. Because the attack characterized here depends on the server altering the shared model graph (inserting a linear imprint probe), the most direct and low-cost safeguard is client-side model-graph integrity verification: before each local training round, clients should validate the received architecture against an expected specification and verify a cryptographic checksum of the model state and its parameter count, rejecting any model whose computation graph or parameter budget deviates. Production FL frameworks such as NVIDIA FLARE, Intel OpenFL, and Owkin Substra already expose client-side hooks and secure-provisioning mechanisms that can enforce this static computation-graph validation and attestation, making the safeguard practical to deploy today. To ensure compliance with HIPAA and GDPR, health care organizations may benefit from running pilot evaluations of these defenses under realistic conditions and document performance trade‐offs. Regulatory bodies could consider developing formal audit protocols for FL systems, encompassing threat modeling, reconstruction testing, and defense efficacy, before granting approval for clinical deployment.

#### Defenses and Practical Mitigations

While this study focused on characterizing gradient inversion risk, effective deployment of federated radiology models requires integrating strong privacy defenses [41]. Common mitigation strategies such as synthetic data generation [42] and DP alone remain insufficient for LLMs [43-45]. Synthetic data can leak statistical artifacts and fail to preserve downstream clinical performance [43,44], whereas DP often requires large privacy budgets to maintain utility, limiting its protective value [46].

In practice, more robust protection arises from hybrid approaches that combine secure aggregation to mask individual updates with task-tuned DP noise, gradient clipping, and auditable privacy monitoring. Cryptographic protocols such as homomorphic encryption or secure multiparty computation offer mathematically proven safeguards but may introduce computational overhead in hospital networks [47]. Future implementations of federated clinical NLP should adopt layered defenses that balance efficiency, regulatory compliance, and measurable privacy guarantees [41,47].

### Limitations

This study was designed as a worst-case evaluation, assuming a fully active malicious server capable of modifying model architecture and exploiting gradient updates. While extreme, this assumption is appropriate for international or cross-consortium federated settings, where governance may be inconsistent and insider threats cannot be ruled out.

Several constraints limit the generalizability of our findings:

1. Dataset scope. We used publicly available radiology corpora (dischargesum and MIMIC-CXR), which lack multimodal components (eg, images), free-text notes, and operational metadata such as timestamps or identifiers. Future evaluations should test gradient inversion on richer, production-like datasets to better estimate clinical leakage risk.
2. Within-batch sequence correlation. Reports were split into nonoverlapping 32-token windows; multiple windows from the same report may co-occur within a single training batch. This violates the IID assumption implicit in our bin-collision analysis and may either inflate or deflate empirical reconstruction success relative to the theoretical per-token collision rate. A correlation-aware extension of the analytic inversion bound is left to future work.
3. Reference-vocabulary overlap scope. The MedGemma NER analysis was applied across all 3 datasets (discharge summaries, diagnostic reports, and MIMIC-CXR) over 5 random seeds (Table 1); overlap was high and statistically indistinguishable across tokenizers in every dataset. Because it is based on exact surface-form matching, it is a lower bound on semantic recovery, and because it is measured against a corpus-level reference vocabulary rather than the specific source report, it is an upper bound on source-aligned recovery.
4. Single reproducible experiment. All reported values—including the paired *t* tests in Multimedia Appendix 4—derive from one reproducible execution of the experimental grid, run under a single fixed configuration with the same random seeds throughout; there is no separate primary or verification experiment. The complete code and per-seed outputs are released, and every reported value can be reproduced by reexecuting the released grid.
5. Retrospective attack focus. This study focused solely on post hoc attack analysis. No privacy-preserving defenses (eg, differential privacy, secure aggregation, and anomaly detection) were implemented or evaluated. While this allows for a clean assessment of leakage potential, it leaves open questions about mitigation feasibility and deployment cost.
6. Tokenizer isolation versus full model effects. We controlled for model architecture to isolate the effect of tokenization on inversion risk. While informative, real-world systems often use both tokenizer and model coadaptation. Thus, privacy risks may be higher or lower depending on the full pipeline design.
7. Data partitioning. Our experiments use an approximately IID partition across 6 simulated clients. Real-world cross-institutional federated deployments are typically non-IID—site-specific protocols, demographic skew, and specialty-specific report distributions can introduce heterogeneity that affects gradient sparsity and consequently inversion vulnerability. The direction of this effect is not a priori clear (non-IID gradients carry stronger per-client signal but also higher variance), and a systematic non-IID evaluation is left for future work.
8. Sequence length. The 32-token regime adopted here is shorter than typical clinical narrative passages. Longer sequences increase the input dimensionality m×L of each per-sample reconstruction, which raises the number of bins k required for unambiguous disentanglement and thereby reduces single-step reconstruction success in practice. Our reported leakage rates are therefore an upper bound for the 32-token regime; longer-sequence regimes are expected to be harder to attack with a single fixed k, all else equal. Quantitatively, the expected per-bin occupancy scales as B×L/k, so an adversary can restore single-occupancy—and hence attack efficacy—by scaling k approximately linearly with L (eg, moving from L=32 to L=512 requires roughly a 16-fold increase in k). This is a linear resource cost rather than an exponential barrier, so longer contexts do not intrinsically prevent closed-form inversion; the practical brake is detectability, since k bins enlarge the injected linear probe by approximately k×(m+1) parameters, making the larger k required for long contexts correspondingly more conspicuous to the parameter-count attestation we recommend below. Extending the analysis to longer-sequence training is left for future work.
9. Client count. Our experiments fix the simulated client count at 6, reflecting a typical small-consortium clinical collaboration. The analytic gradient-inversion attack characterized in this study operates on a single client’s gradient at a single round, and the per-client per-round recovery rate is independent of the number of other clients participating in the federation. Aggregation effects across clients (which arise specifically under cryptographic secure aggregation or weighted averaging schemes that obscure individual client updates) are absent from this evaluation by design and are an explicit target for future defense-oriented work.
10. Probe placement. The imprint module is inserted immediately before the positional embedding so that token-level representations are exposed prior to any token-mixing layer (positional addition and attention). Placement at later layers would conflate tokenizer-segmentation effects with the dynamics of attention and feed-forward mixing, and would not be informative for the central question of this study. A systematic placement ablation across preembedding, postembedding, and postattention positions—holding the tokenizer fixed—would isolate the contribution of placement itself and is identified here as a target for future work.

Taken together, these limitations do not diminish the central contribution highlighting a structural vulnerability in federated clinical NLP but emphasize the need for prospective, defense-integrated evaluations in more realistic medical AI pipelines.

### Future Work

Several extensions to this study are warranted. First, systematic ablation of the imprint module’s placement (preembedding, postembedding, and postattention) would empirically validate the design rationale used here. Second, evaluation of differential privacy as a defense—including utility-privacy trade-off curves at multiple ε values, per-example clipping schedules, and interaction with secure aggregation—is a substantial study in its own right and is the explicit subject of our planned follow-up work. Third, extension of the reference-vocabulary overlap analysis to source-report-aligned entity recovery and to non-IID federated partitions would strengthen the external validity of the leakage estimates reported here.

### Conclusions

FL holds great promise for enabling collaborative, multisite radiology AI without centralizing patient records. However, our gradient-inversion experiments demonstrate that current FL pipelines can leak substantial portions of patient text, raising significant concerns under HIPAA and GDPR standards. In worst-case scenarios, up to approximately 75% of sentences were exactly reconstructed from shared gradients, and approximately 75% of clinical entities, including diagnoses, procedures, and medications, were recovered from the reconstructed text.

Importantly, domain-adapted tokenizers such as RadBERT, while improving semantic fidelity, did not show significantly greater vulnerability to data reconstruction attacks than general-purpose alternatives such as GPT-2 and LLaMA-2. The privacy risk is substantial for all tokenizers and is governed by batch size rather than tokenizer choice.

To ensure safe deployment of federated clinical models, radiology departments and AI developers should rigorously evaluate privacy vulnerabilities in real-world settings, balancing utility with exposure risk. Regulatory bodies and standardization agencies may consider establishing clear audit pathways and certification frameworks tailored to the unique threats posed by clinical text.

## Supplementary material

10.2196/88390 Multimedia Appendix 1 Distribution of exact sentence reconstruction accuracy across datasets, tokenizers, and batch sizes (box plots with the 5 individual runs overlaid).

10.2196/88390 Multimedia Appendix 2 Distribution of recall-oriented understudy for gisting evaluation scores across datasets, tokenizers, and batch sizes (box plots with the 5 individual runs overlaid).

10.2196/88390 Multimedia Appendix 3 95% CIs for exact sentence reconstruction accuracy, computed from the per-cell SDs (Student *t* test, *t*_4_=2.776). Means are reproduced exactly from Multimedia Appendix 1.

10.2196/88390 Multimedia Appendix 4 Pairwise tokenizer paired *t* tests on exact sentence accuracy across the 5 seeds (*df*=4). Δ is the mean per-seed difference in percentage points.

10.2196/88390 Multimedia Appendix 5 Training details and reproducibility configuration. Hyperparameters and infrastructure settings used for all experiments.

10.2196/88390 Multimedia Appendix 6 Exact sentence reconstruction accuracy (%) by tokenizer, dataset, and batch size (mean, SD over 5 seeds).

10.2196/88390 Multimedia Appendix 7 Mean reconstruction scores for sentence-level bilingual evaluation understudy (S) and recall-oriented understudy for gisting evaluation (R) across batch sizes for 3 datasets and tokenizers (mean of 5 seeds).

## Abbreviations

BLEU: bilingual evaluation understudy
DKFZ: German Cancer Research Center
DLG: deep leakage from gradients
DP: differential privacy
FAISS: Facebook AI Similarity Search
FL: federated learning
GDPR: General Data Protection Regulation
HIPAA: Health Insurance Portability and Accountability Act
iDLG: improved deep leakage from gradients
IID: independent and identically distributed
LCS: longest common subsequence
LLM: large language model
MIMIC-CXR: Medical Information Mart for Intensive Care Chest X-Ray
NER: named-entity recognition
NLP: natural language processing
ROUGE-L: recall-oriented understudy for gisting evaluation
S-BLEU: sentence-level bilingual evaluation understudy
