# Supplementary material for: Privacy Leakage in Federated Learning in Radiology Reports: Comparative Evaluation of Tokenizer and Batch-Size Privacy Risks
Source: JMIR Med Inform. 2026 Sep 11;14:e88390. doi: 10.2196/88390 (PMC13568693; doi:10.2196/88390)
Supplement: Supplementary file 3 [file medinform-v14-e88390-s003.docx]

**Multimedia Appendix 3**

| **Tokenizer** | **Dataset** | **Batch** | **Mean (%)** | **95% CI** |
| --- | --- | --- | --- | --- |
| GPT-2 | Discharge | 64 | 64.7 | [60.2, 69.2] |
| GPT-2 | Discharge | 128 | 46.4 | [38.3, 54.5] |
| GPT-2 | Discharge | 256 | 27.3 | [25.3, 29.4] |
| GPT-2 | Diagnosis | 64 | 69.1 | [64.0, 74.1] |
| GPT-2 | Diagnosis | 128 | 47.0 | [45.2, 48.9] |
| GPT-2 | Diagnosis | 256 | 27.2 | [24.7, 29.7] |
| GPT-2 | MIMIC-CXR | 64 | 74.7 | [66.4, 83.0] |
| GPT-2 | MIMIC-CXR | 128 | 49.8 | [46.5, 53.2] |
| GPT-2 | MIMIC-CXR | 256 | 28.7 | [25.4, 31.9] |
| RadBERT | Discharge | 64 | 70.0 | [61.5, 78.5] |
| RadBERT | Discharge | 128 | 50.8 | [48.5, 53.1] |
| RadBERT | Discharge | 256 | 28.5 | [26.3, 30.7] |
| RadBERT | Diagnosis | 64 | 67.5 | [60.9, 74.1] |
| RadBERT | Diagnosis | 128 | 46.4 | [42.4, 50.4] |
| RadBERT | Diagnosis | 256 | 26.6 | [23.5, 29.7] |
| RadBERT | MIMIC-CXR | 64 | 67.5 | [63.3, 71.7] |
| RadBERT | MIMIC-CXR | 128 | 48.9 | [45.4, 52.4] |
| RadBERT | MIMIC-CXR | 256 | 28.8 | [25.5, 32.1] |
| LLaMA-2 | Discharge | 64 | 67.5 | [61.8, 73.2] |
| LLaMA-2 | Discharge | 128 | 48.4 | [45.5, 51.3] |
| LLaMA-2 | Discharge | 256 | 27.5 | [25.6, 29.4] |
| LLaMA-2 | Diagnosis | 64 | 65.9 | [59.0, 72.9] |
| LLaMA-2 | Diagnosis | 128 | 50.2 | [45.5, 54.8] |
| LLaMA-2 | Diagnosis | 256 | 27.6 | [23.0, 32.1] |
| LLaMA-2 | MIMIC-CXR | 64 | 68.4 | [59.7, 77.2] |
| LLaMA-2 | MIMIC-CXR | 128 | 49.5 | [43.7, 55.3] |
| LLaMA-2 | MIMIC-CXR | 256 | 29.5 | [26.6, 32.4] |
