# Supplementary material for: Privacy Leakage in Federated Learning in Radiology Reports: Comparative Evaluation of Tokenizer and Batch-Size Privacy Risks
Source: JMIR Med Inform. 2026 Sep 11;14:e88390. doi: 10.2196/88390 (PMC13568693; doi:10.2196/88390)
Supplement: Supplementary file 4 [file medinform-v14-e88390-s004.docx]

**Multimedia Appendix 4**

| **Batch** | **Dataset** | **Pair** | **Δ (pp)** | **t** | **p** | **Sig** |
| --- | --- | --- | --- | --- | --- | --- |
| 64 | Discharge | GPT-2 vs LLaMA-2 | −2.81 | −1.15 | ≥.05 | ns |
| 64 | Discharge | GPT-2 vs RadBERT | −5.31 | −1.53 | ≥.05 | ns |
| 64 | Discharge | LLaMA-2 vs RadBERT | −2.50 | −0.53 | ≥.05 | ns |
| 64 | Diagnosis | GPT-2 vs LLaMA-2 | +3.12 | 1.10 | ≥.05 | ns |
| 64 | Diagnosis | GPT-2 vs RadBERT | +1.56 | 0.44 | ≥.05 | ns |
| 64 | Diagnosis | LLaMA-2 vs RadBERT | −1.56 | −0.46 | ≥.05 | ns |
| 64 | MIMIC-CXR | GPT-2 vs LLaMA-2 | +6.25 | 1.77 | ≥.05 | ns |
| 64 | MIMIC-CXR | GPT-2 vs RadBERT | +7.19 | 2.82 | <.05 | * |
| 64 | MIMIC-CXR | LLaMA-2 vs RadBERT | +0.94 | 0.26 | ≥.05 | ns |
| 128 | Discharge | GPT-2 vs LLaMA-2 | −2.03 | −0.75 | ≥.05 | ns |
| 128 | Discharge | GPT-2 vs RadBERT | −4.38 | −1.23 | ≥.05 | ns |
| 128 | Discharge | LLaMA-2 vs RadBERT | −2.34 | −1.79 | ≥.05 | ns |
| 128 | Diagnosis | GPT-2 vs LLaMA-2 | −3.12 | −2.14 | ≥.05 | ns |
| 128 | Diagnosis | GPT-2 vs RadBERT | +0.62 | 0.39 | ≥.05 | ns |
| 128 | Diagnosis | LLaMA-2 vs RadBERT | +3.75 | 1.32 | ≥.05 | ns |
| 128 | MIMIC-CXR | GPT-2 vs LLaMA-2 | +0.31 | 0.10 | ≥.05 | ns |
| 128 | MIMIC-CXR | GPT-2 vs RadBERT | +0.94 | 0.91 | ≥.05 | ns |
| 128 | MIMIC-CXR | LLaMA-2 vs RadBERT | +0.62 | 0.23 | ≥.05 | ns |
| 256 | Discharge | GPT-2 vs LLaMA-2 | −0.16 | −0.14 | ≥.05 | ns |
| 256 | Discharge | GPT-2 vs RadBERT | −1.17 | −2.45 | ≥.05 | ns |
| 256 | Discharge | LLaMA-2 vs RadBERT | −1.02 | −1.02 | ≥.05 | ns |
| 256 | Diagnosis | GPT-2 vs LLaMA-2 | −0.39 | −0.29 | ≥.05 | ns |
| 256 | Diagnosis | GPT-2 vs RadBERT | +0.55 | 0.44 | ≥.05 | ns |
| 256 | Diagnosis | LLaMA-2 vs RadBERT | +0.94 | 0.40 | ≥.05 | ns |
| 256 | MIMIC-CXR | GPT-2 vs LLaMA-2 | −0.86 | −0.42 | ≥.05 | ns |
| 256 | MIMIC-CXR | GPT-2 vs RadBERT | −0.16 | −0.10 | ≥.05 | ns |
| 256 | MIMIC-CXR | LLaMA-2 vs RadBERT | +0.70 | 0.46 | ≥.05 | ns |

*1 of 27 pairwise comparisons is significant at α = 0.05 (raw); after Holm correction for the 27 comparisons, none remains significant (all adjusted P = 1.00) — consistent with no systematic tokenizer effect (expected false positives ≈ 1.35 at this α). A three-way variance decomposition of exact-sentence accuracy attributed 94% of variance to batch size and less than 1% to tokenizer.*
