# Supplementary material for: Privacy Leakage in Federated Learning in Radiology Reports: Comparative Evaluation of Tokenizer and Batch-Size Privacy Risks
Source: JMIR Med Inform. 2026 Sep 11;14:e88390. doi: 10.2196/88390 (PMC13568693; doi:10.2196/88390)
Supplement: Supplementary file 5 [file medinform-v14-e88390-s005.docx]

**Multimedia Appendix 5**

| **Parameter** | **Value** |
| --- | --- |
| Optimizer | AdamW |
| Learning rate | 5 × 10⁻⁴ |
| Local batch size (sequences) | 64, 128, 256 |
| Sequence length | 32 tokens |
| FL rounds per measurement | 1 (single-step attack per gradient-inversion measurement) |
| Aggregation rule | FedAvg (logical; per-client per-round inversion) |
| Simulated clients | 6 (uniform random partition; IID) |
| Independent runs per configuration | 5 (different random seeds) |
| Foundation model | 3-layer Transformer encoder; m = 96, 8 heads, FFN 1536; ≈ 13.4 M parameters |
| Tokenizers | GPT-2 (50,257); StanfordAIMI RadBERT (30,522); LLaMA-2-7B (32,000) |
| Imprint bins (K) | 1,000 |
| Imprint linear-function init | Random Gaussian (linfunc = randn), gain 1.0 |
| Imprint bias init | Cumulative Laplace quantile thresholds |
| Numerical stability ridge ε | 10⁻⁸ |
| Hardware | NVIDIA A100 (40 GB), single-GPU per run |
| Software | Python 3.10, PyTorch 2.x, HuggingFace Transformers, breaching library |
| Random seeds | {0, 1, 2, 3, 4} |
