# Supplementary material for: Privacy Leakage in Federated Learning in Radiology Reports: Comparative Evaluation of Tokenizer and Batch-Size Privacy Risks
Source: JMIR Med Inform. 2026 Sep 11;14:e88390. doi: 10.2196/88390 (PMC13568693; doi:10.2196/88390)
Supplement: Supplementary file 6 [file medinform-v14-e88390-s006.docx]

**Multimedia Appendix 6**

| **Tokenizer (vocab)** | **Dataset** | **Batch 64** | **Batch 128** | **Batch 256** |
| --- | --- | --- | --- | --- |
| GPT-2 (50,257) | Discharge | 64.7 ± 3.6 | 46.4 ± 6.5 | 27.3 ± 1.7 |
|  | Diagnosis | 69.1 ± 4.0 | 47.0 ± 1.5 | 27.2 ± 2.0 |
|  | MIMIC-CXR | 74.7 ± 6.7 | 49.8 ± 2.7 | 28.7 ± 2.6 |
| RadBERT (30,522) | Discharge | 70.0 ± 6.8 | 50.8 ± 1.8 | 28.5 ± 1.8 |
|  | Diagnosis | 67.5 ± 5.3 | 46.4 ± 3.2 | 26.6 ± 2.5 |
|  | MIMIC-CXR | 67.5 ± 3.4 | 48.9 ± 2.8 | 28.8 ± 2.7 |
| LLaMA-2 (32,000) | Discharge | 67.5 ± 4.6 | 48.4 ± 2.3 | 27.5 ± 1.5 |
|  | Diagnosis | 65.9 ± 5.6 | 50.2 ± 3.8 | 27.6 ± 3.7 |
|  | MIMIC-CXR | 68.4 ± 7.0 | 49.5 ± 4.7 | 29.5 ± 2.3 |

*All values derive from a single reproducible experimental grid: 3 tokenizers × 3 datasets × 3 batch sizes × 5 random seeds = 135 gradient-inversion attack runs, executed under one fixed configuration. Code and per-seed outputs are released (see Data Availability).*
