# Supplementary material for: Privacy Leakage in Federated Learning in Radiology Reports: Comparative Evaluation of Tokenizer and Batch-Size Privacy Risks
Source: JMIR Med Inform. 2026 Sep 11;14:e88390. doi: 10.2196/88390 (PMC13568693; doi:10.2196/88390)
Supplement: Supplementary file 7 [file medinform-v14-e88390-s007.docx]

**Multimedia Appendix 7**

| **Dataset** | **Tokenizer** | **64 (S / R)** | **128 (S / R)** | **256 (S / R)** |
| --- | --- | --- | --- | --- |
| Discharge | GPT-2 | 0.66 / 0.67 | 0.49 / 0.50 | 0.31 / 0.33 |
|  | RadBERT | 0.71 / 0.72 | 0.53 / 0.54 | 0.32 / 0.34 |
|  | LLaMA-2 | 0.69 / 0.69 | 0.51 / 0.52 | 0.30 / 0.32 |
| Diagnosis | GPT-2 | 0.70 / 0.71 | 0.49 / 0.50 | 0.31 / 0.32 |
|  | RadBERT | 0.68 / 0.68 | 0.48 / 0.49 | 0.30 / 0.32 |
|  | LLaMA-2 | 0.67 / 0.67 | 0.52 / 0.53 | 0.31 / 0.33 |
| MIMIC-CXR | GPT-2 | 0.75 / 0.75 | 0.52 / 0.53 | 0.32 / 0.33 |
|  | RadBERT | 0.68 / 0.68 | 0.50 / 0.51 | 0.32 / 0.33 |
|  | LLaMA-2 | 0.70 / 0.70 | 0.51 / 0.52 | 0.32 / 0.33 |

*The G-BLEU (corpus-level BLEU) metric reported in earlier versions has been removed because it is not defined at the per-seed level and therefore cannot be reproduced within this per-seed grid.*
