# Supplementary material for: Privacy Leakage in Federated Learning in Radiology Reports: Comparative Evaluation of Tokenizer and Batch-Size Privacy Risks
Source: JMIR Med Inform. 2026 Sep 11;14:e88390. doi: 10.2196/88390 (PMC13568693; doi:10.2196/88390)
Supplement: Supplementary file 8 [file medinform-v14-e88390-s001.docx]

**Multimedia Appendix 1**
